# Effect of Lactic Acid Bacteria on the Fermentation Quality and Mycotoxins Concentrations of Corn Silage Infested with Mycotoxigenic Fungi

**DOI:** 10.3390/toxins13100699

**Published:** 2021-10-01

**Authors:** Jinyang Li, Wenbo Wang, Sifan Chen, Tao Shao, Xuxiong Tao, Xianjun Yuan

**Affiliations:** Institute of Ensiling and Processing of Grass, College of Agro-Grassland Science, Nanjing Agricultural University, Nanjing 210095, China; 2019120015@njau.edu.cn (J.L.); wwb05240108@163.com (W.W.); 2020220002@stu.njau.edu.cn (S.C.); taoshaolan@163.com (T.S.); 2019220002@njau.edu.cn (X.T.)

**Keywords:** corn silage, microbial community, fungi infestation, mycotoxins

## Abstract

This study was conducted to evaluate the effect of lactic acid bacteria (LAB) on fermentation quality, mycotoxin concentrations, and microbial communities of whole-crop corn silages infested with mycotoxigenic fungi. Cultured spores (10^6^ cfu/mL) of mycotoxigenic *Aspergillus flavus* and *Fusarium graminearum* were sprayed (5 mL) on corn forage on 27 July and 10 August 2018. On 21 August 2018, sprayed (FI; 3 plots) and unsprayed (NFI; 3 plots) corn forage were harvested at the 1/2 kernel milk line stage, followed by chopping and ensiling without inoculants (CON), or with *Lactobacillus buchneri* (LB, 1 × 10^6^ cfu/g FW), *Lactobacillus plantarum* (LT, 1 × 10^6^ cfu/g FW), or *L. buchneri* + *L. plantarum* (BT: both *L. buchneri* and *L. plantarum* applied at 0.5 × 10^6^ cfu/g FW). After 90 d of ensiling, FI silages had a higher (*p* < 0.05) pH value and higher acetic acid (ACA), ethanol, and ammonia nitrogen (ammonia N) concentrations, but lower (*p* < 0.05) lactic acid (LA) concentrations than NFI silage. The inoculants decreased pH and increased LA concentration and LA/ACA compared with CON. The aflatoxin B_1_ (AFB_1_) was only detected in FI fresh corn and silages; ensiling decreased (*p* < 0.05) AFB_1_ concentration compared with fresh corn, and LB and BT decreased AFB_1_ concentration compared with CON. The zearalenone (ZEN), deoxynivalenol (DON), and fumonisin B_1_ (FB_1_) concentrations were similar (*p* < 0.05) for NFI silages, while ZEN concentration in BT was the lowest (*p* < 0.05) among all FI silages; DON and FB_1_ concentrations in LB, LT, and BT silages were significantly lower (*p* < 0.05) than those of CON in FI silages. The fungal infestation increased the bacterial and fungal diversity of silages compared with NFI silages. The FI silages had a higher relative abundance (RA) of *Lactobacillus*, *Weissella*, *Wickerhamomyces*, *Pichia*, and *Epicoccum* than the corresponding NFI silages. The RA of *Aspergillus* and *Fusarium* markedly decreased after 90 d of ensiling, and the inoculation expanded this trend irrespective of fungal infestation. The *Penicillium* in FI silages survived after 90 d of ensiling, while the inoculants decreased the RA of *Penicillium*. Inoculants mitigate the adverse effects of fungal infestation on corn silage quality by changing the bacterial and fungal communities.

## 1. Introduction

Warm and humid conditions favor rust development, which commonly occurs in tropical and subtropical regions. Rust infestations in corn damage the leaf cuticle, resulting in an increase of concentrations of dry matter (DM), neutral detergent fiber (NDF), and acid detergent fiber (ADF) and a decrease of biomass yields and DM digestibility of forage. More importantly, rust infestations predispose corn to mycotoxin contamination, which is a serious threat to dairy operations that feed corn silage. Queiroz et al. [1] reported that increasing rust infestation was associated with reductions in digestibility and fermentation quality of corn silages, while aflatoxin was only detected in high levels of southern rust-infested corn silages, and they also found that the inoculation reduced adverse effects of rust infestation on the fermentation and aflatoxin production in heavily rust-infested corn silages. Jensen et al. [2] assessed the effect of the ensiling process on the fate of *Fusarium* mycotoxins of forage maize and found that ensiling is not a practical method for reducing *Fusarium* mycotoxins present at harvest; however, Ma et al. [3] reported that, regardless of lactic acid bacteria (LAB) inoculation, certain silage bacteria could reduce the spiked concentration of aflatoxin B_1_ (AFB_1_) in corn silage to a safe level. Nevertheless, no study has been conducted to reveal how the epiphytic or inoculated bacteria interact with epiphytic or infested fungi during ensiling. Novel culture-independent techniques, such as next-generation sequencing (NGS), can improve our understanding of silage microbial communities. Revealing the dynamics of bacterial and fungal communities during ensiling of rust-infested corn is critical to alleviate the adverse effects of fungal infestation on the fermentation and hygienic quality of silages in tropical and subtropical regions.

Eliminating mycotoxins in food or feed has been increasingly attracting the concern of health organizations. Many efforts to limit the production of mycotoxins in silage during the field, pre-harvest, harvest, and ensiling phases have been undertaken [4]. Of these strategies, biological decontamination and biodegradation with microorganisms or enzymes are attractive solutions to eliminate mycotoxins. Many species of bacteria and fungi have been shown to enzymatically degrade mycotoxins, which are potentially promising candidates to detoxify mycotoxins in feed and foods due to irreversibly catalyzed reactions.

Bacteria, yeast, and fungi with detoxification abilities have been isolated from different sources, and LAB are the preferred candidates for eliminating mycotoxins in silages because they play a critical role in the ensiling fermentation. *Lactobacillus plantarum* and *Lactobacillus buchneri* are known to enhance lactic acid (LA) fermentation and acetic acid (ACA) production to improve fermentation quality and aerobic spoilage. Ma et al. [3] reported that some strains of LAB in *L. plantarum*, *L. buchneri*, and *P. acidilactici* species can bind to AFB1 in vitro. However, no study has evaluated their effects on mycotoxin concentrations of corn silages, and it is still unclear how the inoculants interact with infested fungi during the ensiling of corn. It is hypothesized that the fungal infestation would detrimentally affect the silage fermentation and hygienic quality of corn silage, and inoculating *L. plantarum* and *L. buchneri* would mitigate potential negative effects arising from fungal infestation. Thus, our objective was to determine the effects of LAB inoculants on the fermentation, microbial communities, and mycotoxin concentrations of corn silage contaminated with toxigenic fungi.

## 2. Results

### 2.1. Chemical and Microbial Composition of Fresh Corn

The DM, water-soluble carbohydrates (WSC), crude protein (CP), NDF, ADF, starch concentrations, and microbial populations of fresh corn are shown in Table 1. There were no significant (*p* > 0.05) differences in the concentrations of DM, WSC, CP, NDF, ADF, starch, and the populations of LAB, aerobic bacteria (AB), and *Enterobacteria* between the fresh FI and NFI corn prior to ensiling. The populations of yeasts and molds in FI corn were higher (*p* < 0.05) than those of the NFI corn before ensiling.

### 2.2. Fermentation Characteristics of Whole-Crop Corn Silages

The pH, WSC, fermentation products, and microbial populations of corn silages after 90 d of ensiling are shown in Table 2. There was an interaction (*p* < 0.01) between fungal infestation and inoculants for pH, because the pH in LB was similar to that of CON in FI silages (*p* < 0.05, 3.64 vs. 3.66), while the pH in LB, LT, and BT was significantly lower than that of CON in NFI silages, respectively (*p* < 0.05). There was no interaction (*p* > 0.01) between fungal infestation and inoculants for LA and ACA concentrations; however, both fungal infestation and inoculants significantly affected LA and ACA concentrations (*p* < 0.01). The LA concentration in NFI silage was higher than that of FI silage (*p* < 0.05, 101 vs. 92.5 g/kg DM); the inoculants significantly increased the LA concentration compared with CON silages (*p* < 0.05). The ACA concentration in FI silage was higher than that of NFI silage (*p* < 0.05, 28.4 vs. 15.9 g/kg DM); the ACA concentration in LT was significantly lower than that of LB silages (*p* < 0.05, 21.3 vs. 23.5 g/kg DM). An interaction between fungal infestation and inoculants for LA/ACA was observed because the inoculants increased (*p* < 0.05) the LA/ACA for NFI silage, while there was no difference (*p* > 0.05) in LA/ACA for FI silage. The ethanol concentration was significantly affected by fungal infestation (*p* < 0.01) with a higher value in FI silage (23.2 g/kg DM) than NFI silage (20.3 g/kg DM). The LB silages (32.6 g/kg DM) showed a significantly lower WSC concentration than CON silages (40.0 g/kg DM). Both fungal infestation (*p* < 0.01) and inoculants (*p* = 0.01) significantly affected the ammonia N concentration, indicated by a higher ammonia N concentration in FI silage (107 g/kg TN) than NFI silage (71.0 g/kg TN). The ammonia N concentration in BT was similar (*p* > 0.05) to that of LB and LT silages, but lower (*p* < 0.05) than that of CON. There was no interaction between fungal infestation and inoculants for the populations of yeast and LAB (*p* > 0.01). There was an interaction between fungal infestation and inoculants for the population of AB (*p* < 0.05).

### 2.3. The Concentrations of AFBs, ZEN, DON, and FBs of Whole-Crop Corn Silages

The mycotoxin concentrations of corn silages after 90 d of ensiling are shown in Table 3. There was an interaction (*p* < 0.01) between fungal infestation and inoculants for the concentration of AFB_1_ because the AFB_1_ in NFI fresh corn and silages was below the detection levels, while the AFB_1_ concentration in LB and BT silages was lower (*p* < 0.05) than that of CON in FI silages. For the FI silages, ensiling decreased (*p* < 0.05) the AFB_1_ concentration compared with fresh corn prior to ensiling. There was an interaction (*p* < 0.01) between fungal infestation and inoculants for concentrations of ZEN, DON, and FB_1_ because ZEN, DON, and FB_1_ concentrations were similar (*p* < 0.05) for NFI silages, while ZEN concentration in BT was the lowest (*p* < 0.05) among FI silages. The DON and FB_1_ concentrations in LB, LT, and BT silages were significantly lower (*p* < 0.05) than those of CON in FI silages.

### 2.4. Bacterial and Fungal Composition of Whole-Crop Corn Silages

Sequencing bacterial 16S rRNA V_3_–V_4_ regions resulted in 2,594,761 reads, with an average of 48,051 quality sequence reads per sample after quality filtering. All of these quality reads were clustered into a total of 1184 OTUs based on a 97% sequence identity threshold. The average Good’s coverage for all samples was greater than 99%, indicating that the sequencing depth was adequate for reliable analysis of the bacterial community. Sequencing fungal ITS resulted in 3,100,921 reads, with an average of 57,424 reads per sample after quality filtering. All of these quality reads were clustered into a total of 925 OTUs based on a 97% sequence identity threshold. The average Good’s coverage (Table 4) for all samples was greater than 99%, indicating that the coverage of fungal diversity was sufficient to evaluate the fungal community.

The α-diversity indices of bacterial and fungal communities are shown in Table 4. For the bacterial community, ensiling decreased Chao 1 as compared to fresh corn. FI silage showed a higher (*p* < 0.05) Shannon index than NFI silage. The addition of LT decreased the Chao 1 index. Regarding the fungal community, the fungal infestation decreased the Chao 1 and Shannon indexes of fresh corn prior to ensiling. The FI silage had a higher Shannon index than NFI silage. There was no significant difference in the Chao 1 and Shannon indexes among the four treatments. The Chao 1 and Shannon indexes in 5-day silages were higher than those of 90-day silages.

**Figure 1:**
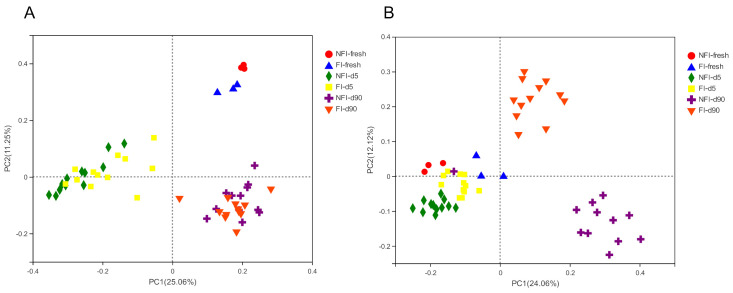
Principal coordinate analysis (PCoA) plot based on unweighted-UniFrac dissimilarity distance of the bacterial ((**A**), R = 0.6911, *p* = 0.001) and fungal ((**B**), R = 0.7088, *p* = 0.001) community between samples. FI, fungal infestation; NFI, non-fungal infestation.

The unweighted principal coordinate analysis UniFrac plot (PCoA, R = 0.6559, *p* = 0.001) showed differences in bacterial communities among the fresh corn, 5-day, and 90-day silages (Figure 1A). The fresh samples were clustered in the first quadrant, the 5-day silages were clustered in the second and third quadrant, while the 90-day silages were separately assigned to the fourth quadrant. Figure 1B (R = 0.6378, *p* = 0.001) shows the compositional differences in fungal communities among the samples. The fresh corn and 5-day silages were clustered together and separated from the 90-day silages, and there was a clear separation between the FI 90-day and NFI 90-day silages.

**Figure 2:**
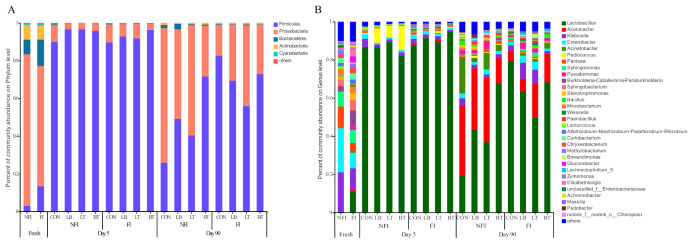
Bacterial community at the phylum (**A**) and genus (**B**) level for corn silage during ensiling. FI, fungal infestation; NFI, non-fungal infestation; CON, control; LB, *L. buchneri* applied at 1 × 10^6^ cfu/g FW; LT, *L. plantarum* applied at 1 × 10^6^ cfu/g FW; BT, *L. buchneri* and *L. plantarum* applied at 0.5 × 10^6^ cfu/g FW.

The succession of the bacterial community on the phylum and genus level during the ensiling is illustrated in Figure 2. Five phyla (*Firmicutes*, *Proteobacteria*, *Bacteroidetes*, *Actinobacteria*, and *Cyanobacteria*) were detected in all samples. *Proteobacteria* was the most predominant phylum in fresh FI (62.13%) and NFI (80.33%) corn, while *Firmicutes* became the most predominant phylum after 5 d of ensiling for all silages.

There were 18 and 15 genera with an RA greater than 1% in fresh FI and NFI corn, respectively (Figure 2B). *Klebsiella* (11.43%) was the most abundant genus in fresh FI corn, followed by *Lactobaccillus* (10.99%) and *Enterobacter* (8.06%). *Enterococcus* (23.03%) was the most predominant genus in NFI corn prior to ensiling, followed by *Klebsiella* (20.83%), *Pediococcus* (11.27%), and *Sphingomonas* (7.71%).

*Lactobacillus* became the most predominant bacterial genus in all silages after 5 d of ensiling, and they remained having the predominant role in 90-day silages, except CON in NFI silages, although there was a slight decline in the RA of *Lactobacillus* in all silages. The FI silage always had a higher RA of *Lactobacillus* than the corresponding NFI silage. *Acetobacter* was detected in all 90-day silages. The NFI silage had a higher RA of *Acetobacter* than the corresponding FI silage, and inoculants decreased the RA of *Acetobacter* compared with CON for NFI silages. *Klebsiella* was rapidly eliminated after 5 d of ensiling, and the inoculants decreased the RA of *Klebsiella* in FI 5-day silages as well as NFI 5-day and 90-day silages. The RA of *Enterobacter* in all silages decreased below 5% of the total sequence after 5 d of ensiling. *Acinetobacter* was not detected after 5 d of ensiling; however, it appeared in all 90-day silages. For NFI silages, the inoculants decreased the RA of *Acinetobacter* as compared with CON (19.1%). *Pediococcus* and *Lactococcus* were mainly detected in NFI 5-day silages. The inoculants increased the RA of *Pediococcus* to greater than 5% in the NB, NT, and NBT of 5-day silages, while they decreased the RA of *Lactococcus*. *Pantoea* decreased below 2% of the total sequence after 5 d of ensiling. *Sphingomonas* and *Pseudomonas* were only detected after 90 d of ensiling. The FI silage had a higher RA of *Weissella* than the NFI silage, regardless of the ensiling days. *Zymononas* was detected in FI 5-day silages, and the inoculants decreased the RA of *Zymononas*, then disappeared in all 90-day silages.

**Figure 3:**
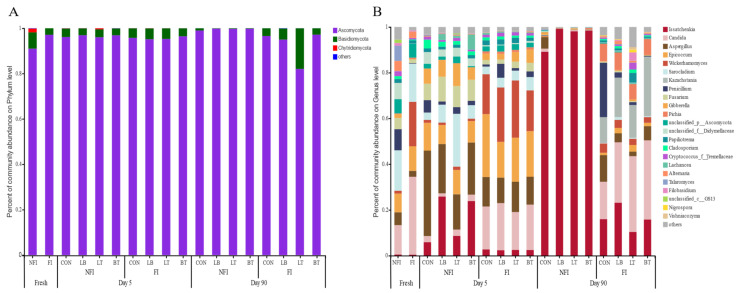
Fungal community at the phylum (**A**) and genus (**B**) level for corn silage during ensiling. FI, fungal infestation; NFI, non-fungal infestation; CON, control; LB, *L. buchneri* applied at 1 × 10^6^ cfu/g FW; LT, *L. plantarum* applied at 1 × 10^6^ cfu/g FW; BT, *L. buchneri* and *L. plantarum* applied at 0.5 × 10^6^ cfu/g FW.

The succession of the fungal community on the phylum and genus level during ensiling of corn is illustrated in Figure 3. Three phyla (*Asconycota*, *Basidiomycota*, and *Chytridiomycota*) were detected in fresh corn prior to ensiling, and *Asconycota* was the most predominant phylum in samples irrespective of ensiling. *Chytridiomycota* was not detected in corn silages. There were 11 and 16 genera each with an RA greater than 1% in fresh FI and NFI corn, respectively. *Sacocladium* (17.63%) was the most abundant genus in fresh FI corn, followed by *Candida* (12.83%) and *Penicillium* (9.28%)*. Candida* (34.05%) was the most predominant genus in fresh NFI corn, followed by *Wickerhamomyces* (19.46%), *Sacocladium* (16.69%), and *Epicoccum* (10.74%).

The RA of *Issatchenkia* increased after ensiling, and its RA in NFI silage was higher than that of the corresponding FI silage (Figure 3B). For NFI silages, the inoculants increased the RA of *Issatchenkia* regardless of the number of ensiling days. The RAs of *Candida* and *Wickerhamomyces* in the fresh FI corn and FI silages were always higher than those of the fresh NFI corn and NFI silages. The RA of *Aspergillus* markedly decreased after 90 d of ensiling, and the inoculants decreased the RA of *Aspergillus* compared with CON silages irrespective of the fungal infestation. The RAs of *Epicoccum* and *Pichia* in fresh NFI corn and NFI silages were always lower than those of the corresponding fresh FI corn and FI silages.

*Sarocladium*, *Fusarium*, and *Gibberella* were detected in the fresh corn and 5-day silages and disappeared after 90 d of ensiling. *Kazachstania* was only detected in the FI 90-day silages. For the NFI 5-day silages and FI 90-day silages, the inoculants decreased the RA of *Penicillium*. The *unclassified_f_Didymellaceae* was mainly detected in the fresh corn and 5-day silages of NFI, and it almost disappeared after 90 d of ensiling.

## 3. Discussion

### 3.1. Effect of Inoculants on Fermentation Quality of Corn with or without Fungal Infestation

After 90 d of ensiling, the pH in all silages ranged between 3.44 and 3.66, indicating that the silage fermentation was satisfactory regardless of fungal infestation. The FI silages had a higher silage pH, which was accompanied with a lower LA concentration in the FI silage than the NFI silage. The fungal infestation decreased the fermentation efficiency and delayed acidification because of the loss of critical fermentation substrates and the flourish of more bacteria. Hassani et al. [5] reported that the infestation of *Puccinia* spp. to corn can convert photosynthates, such as glucose and fructose, into other fungus-specific products, and they can thrive at high-pH conditions during ensiling.

In the study, LT alone or in combination with LB increased the LA concentration and decreased the pH values compared with CON silages regardless of fungal infestation. This was due to the faster and greater production of organic acids by the fermentation of inoculated LAB, which rapidly dominated the ensiling. In previous studies, inoculating *L. plantarum* or *L. buchneri* reduced the time required for the elimination of *Escherichia coli* in alfalfa silages because they accelerated the accumulation of lactate and the reduction in pH during ensiling [6].

The higher ACA concentration in the FI silage than the NFI silage was related to a higher RA of *Weissella* in FI silage than that of NFI silage. Bacteria assigned to the genus *Weissella* are strictly heterofermentative, producing a mixture of lactate and acetate as the major end products of sugar metabolism. Cai et al. [7], inoculating *Weissella paramesenteroides* to alfalfa silage, increased the acetate concentration. Correspondingly, the NFI silage had higher LA/ACA than the FI silage. The higher ACA concentration in LB silages than LT silages was attributed to its heterofermentative pathway, which fermented hexoses or pentoses to lactic acid, acetic acid, and/or ethanol. In NFI silages, inoculating LAB increased the LA/ACA ratio compared with CON; however, there was no significant difference in LA/ACA among treatments in FI silages, and this was attributed to the high ACA production in FI silages as mentioned above. Although we did not detect the ethanol concentration of fresh corn before ensiling, the higher ethanol concentration in the FI silage than the NFI silage might be related to the converting of photosynthates to alcohols by infested fungi before ensiling [8].

In the study, we found a lower WSC concentration in LB silages than CON; Kleinschmit and Kung [9] reported that the lower amount of WSC in silages inoculated with *L. buchneri* was due to the consumption of sugars by the inoculants of *L. buchneri*.

During infection, phytopathogenic fungi must obtain enough nitrogen from their plant hosts to promote fungal fitness, survival, and virulence within the host; they have developed sophisticated mechanisms for nitrogen acquisition, and ammonium and glutamine are the preferred nitrogen sources [10]. Thus, more available ammonia N were detected in the FI silage than the NFI silage. The inoculants enhanced the organic acid production and pH decline, which inhibited the proteolysis activity of plant intrinsic protease and undesirable microorganisms, which resulted in lower ammonia N in the inoculated silages than CON [11].

### 3.2. Effect of Inoculants on the AFB_S_, ZEN, DON, and FBs Concentrations of Corn Silage with or without Fungal Infestation

The AFB_1_ and AFB_2_ were only detected in FI fresh corn and silages, although both of them in FI silages were below the maximum permissible levels of AFB_1_ and AFB_2_ of 50 ug/kg stipulated in China for the dietary ingredients of dairy cattle [12]. Queiroz et al. [1] also reported that aflatoxin was only detected in high levels of rust-infested corn silages.

The concentration of AFB_1_ decreased after 90 d of ensiling compared with the fresh corn. Garon et al. [13] observed a decrease in AFB_1_ concentration of farm-scale silages as the ensiling duration increased. High levels of aflatoxins in corn silage are notable but not unique. Silage is expected to have low concentrations of aflatoxins because aflatoxins are primarily produced by *A. flavus* and *A. parasiticus*, which have a low tolerance to anaerobic conditions and low pH in silages [14]. Compared with CON, the lower AFB_1_ concentration in LB and BT silages for the FI silages suggested that inoculation prevented the accumulation of the aflatoxins in the silages. This could be attributable to a detoxification effect of inoculants during ensiling, as reported by Oluwafemi et al. [15], who observed that some strains of LAB were able to degrade AFB_1_ in corn grain via a biological pathway. Ahlberg et al. [16] compared the ability to bind aflatoxins among different LAB species in different food matrices and found that their binding effects ranged from 0% to 90%. Ma et al. [3] observed the capacity of binding AFB_1_ in vitro by some strains of LAB when applied at 10^9^ cfu/mL. Those authors failed to find the similar effect on corn silage, although they concluded that some silage bacteria could reduce the AFB_1_ to a safe concentration within 3 d of ensiling, regardless of LAB inoculation.

Relative to NFI fresh corn, the fungi infestation increased the concentrations of *Fusarium* toxins including ZEN, FBs, and DON, which produced by several species of *Fusarium* such as *F. graminearum*, *F. roseum*, *F. culmorum*, and *F. crookwellense*. The presence of ZEN, FBs, and DON in NFI fresh corn and silages indicated that the corns were infected by *Fusarium* spp. before harvest; this was attributed to the high-humidity conditions with moderate temperatures in Southern China, which favored the growth of *Fusarium* spp.

Ensiling decreased concentrations of *Fusarium* toxins compared with fresh corn regardless of fungal infestation. All inoculants decreased the DON and FB_1_ concentrations, while only BT decreased the ZEN concentration compared to CON in FI silages. Several bacteria showed a detoxification capacity of *Fusarium* toxins, and LAB are the preferred candidates because of their essential role in silage. Niderkorn et al. [17] compared 202 strains for the ability to bind and/or biotransform *Fusarium* toxins and found that eight *Lactobacilli* and three *Leuconostoc* biotransformed ZEN into α-zearalenol, and most strains could bind DON, ZEN, and FBs. The ability to bind *Fusarium* toxins seems to be a common property for fermentative bacteria, which helps to explain the decline of ZEN and DON after 90 d of ensiling. Vandicke et al. [18] indicated that the mycotoxin contamination of maize silages largely originates from the initial contamination before ensiling, but the levels of mycotoxins are generally reduced throughout the ensiling process, and they hypothesized that the mycotoxin concentrations decreased by elution, degradation, or adsorption during ensiling. Vega et al. [19] demonstrated that a *L. plantarum* isolated from pigs has great potential to remove zearalenone through absorption. However, how the ensiling process reduces mycotoxin concentrations is still up to debate. Thus, the metabolite analysis or adsorption assay should be conducted to confirm the mechanism of *L. buchneri* and *L. plantarum* on eliminating mycotoxins in the future.

### 3.3. Effect of Inoculants on Bacterial and Fungal Diversity and Composition of Corn with or without Fungal Infestation

Relative to fresh corn, ensiling decreased the bacterial diversity regardless of ensiling days. Zheng et al. [20] attributed the lower bacterial richness and diversity in the silage than fresh alfalfa to the disappearance of some epiphytic bacteria because of their lower adaptability to the anaerobic and acidic conditions.

The higher Shannon diversity index in the FI silage than the NFI silage was related to the higher pH during ensiling. Mendez-Garcia et al. [21] reported that low pH was the main factor underlying the limited microbial diversity during ensiling. Inoculating LAB increased the RA of the predominant genus, reducing the bacterial alpha diversity of silages because, the greater the abundance of dominant bacteria, the less diverse the microbial community. Ogunade et al. [6] reported that *L. plantarum* and *L. buchneri* added to alfalfa outcompeted other bacteria or inhibited the undesirable bacteria by reducing the pH, resulting in the reduction in bacterial diversity.

The clear separation among the fresh corn, 5-day, and 90-day silages in PCoA plots indicated the differences in the distribution and structure of bacterial community between fresh corn and silages. The separation of silages from fresh material was attributed to the suppression or inactivation of some epiphytic bacteria species during the ensiling. All 90-day silages were clustered together regardless of fungal infestation, showing that similar bacterial communities were formed after 90 d of ensiling. Consistent with the study of Zheng et al. [20], the richness of the bacterial community decreased at the early stage of ensiling of wilted alfalfa, whereas it flourished on day 90 of ensiling.

*Proteobacteria* was the most predominant phylum in fresh corn regardless of fungal infestation, while the dominant phyla present in silages was *Firmicutes*. This observation was corroborated by Romero et al. [22], who reported that *Proteobacteria* was the dominate phylum (84%) in fresh corn, while *Firmicutes* became the most abundant phylum (86.0%) in whole-crop corn ensiled for 100 d. *Xanthomonadales*, *Pseudomonadales*, *Enterobacteriales*, and *Sphingomonadales*, belonging to *Proteobacteria*, were usually found in standing crops; however, they are not the desirable bacteria for ensiling fermentation. *Firmicutes*, represented by genera of *Lactobacillus*, *Lactococcus*, and *Weissella*, are important acid hydrolytic microorganisms in anaerobic environments.

In fresh corn, more genera with an RA greater than 1% were present in FI corn than NFI corn prior to ensiling. The artificial fungal infestation in corn caused the change in the interaction strategies between endophytic microbes and the plant host, resulting in changes of the phyllosphere microbial community of corn.

*Klebsiella* was the most abundant genus in FI corn before ensiling. The *Klebsiella* genus, belonging to the *Enterobacteriaceae* family, can be found in a variety of plant hosts. In the study, more *Klebsiella* was detected in fresh FI corn than fresh NFI corn (23.03% vs. 11.43%). Fouts et al. [23] reported that the high RA of *Klebsiella* in plants was partly attributable to their lack of a flagellum, as flagella are known to induce plant defenses. The fungal infestation induced the plant defenses, which in turn reduced the numbers of *Klebsiella*.

*Lactobacillus* became the most predominant bacterial genus in all silages after 5 d of ensiling, and remained predominant in 90-day silages. The genus *Lactobacillus* is one of the most important genera for ensiling fermentation; these bacteria are found in a wide variety of habitats and have been used in the manufacture of fermented foods, as well as animal feeds. Yuan et al. [24] found that *Lactococcus* was the predominant genus during the initial 3 d of ensiling of Napier grass; however, its dominant role was gradually replaced by *Lactobacillus* because of the stronger acid tolerance of *Lactobacillus* than lactic acid-producing cocci. The FI silages always had a higher RA of *Lactobacillus* than the corresponding NFI silages. The lower bacterial diversity and unstable bacterial community in the FI corn might be more conducive to establishing a dominant role of *Lactobacillus* during ensiling.

*Acetobacter* is ubiquitous in plants and is recognized as ACA-producing bacteria, which partly contribute to the pH decline at the early stage of ensiling. Guan et al. [25] evaluated the effect of heat-resistant LAB on the microbial community and fermentation dynamics of corn silage in a hot environment and found that *Acetobacter* could also be found in well-sealed small-scale silage, and its presence was associated with high temperatures. This is in accordance with the present study, *Acetobacter* was detected in all of 90-day silages.

The occurrence of *Acinetobacter* is undesirable in silages, since it has been linked to aerobic deterioration and the DM losses [26]. In the study, *Acinetobacter* was detected in all 90-day NFI silages, and the inoculations decreased the RA of *Acinetobacter* as compared with CON (19.1%), which might be attributed to the lower pH. Ogunade et al. [26] reported that *L. buchneri*-treated silage had greater RAs of *Acinetobacter* and *Weissella* than the control silage, which resulted from the high acetate concentration, because *Acinetobacter* can survive in an anaerobic environment in the presence of acetate as a substrate.

*Pediococcus* and *Lactococcus* were mainly detected in the NFI 5-day silages; the inoculation increased the RA of *Pediococcus* to above 5% in the NB, NT, and NBT of the 5-day silages, while it decreased the RA of *Lactococcus*. *Pediococcus* genera are usually used as silage inoculants due to their tolerance to high DM and pH conditions, allowing them to dominate the initial stages of fermentation. *Lactococci* grow best at near-neutral pH conditions but cease to grow at about pH 4.5; in the present study, the inoculants enhanced the LA fermentation and accelerated the pH decline and advanced the cease of *Lactococci* before day 5. Romero et al. [27] reported that adding *L. buchneri* resulted in silages dominated by the *Lactobacillus* genus with much less diversity than the untreated silages, which sustained more other genera.

*Pantoea* decreased below 2% of total sequence after 5 d of ensiling; *Sphingomonas* and *Pseudomonas* were only detected after 90 d of ensiling. Keshri et al. [28] found that the RA of *Pantoea* immediately decreased after 5 h of ensiling. *Pantoea*, belonging to the *Enterobacteriaceae* family, is undesirable for ensiling fermentation because it can utilize LA, causing nutrient loss.

The higher RA of *Weissella* in FI silages than NFI silages regardless of ensiling days might be attribute to antifungal activity. Ndagano et al. [29] reported that two strains belonging to *Weissella* genus showed antifungal activities because they can produce antifungal compounds such as 2-hydroxy-4-methylpentanoic acid. Baek et al. [30] indicated that *W. confusa* as a starter strain was the dominating species in rice dough fermentation and delayed the rapid growth of fungal contaminants such as *Penicillium crustosum*, due to the high concentrations of organic acids including acetic and lactic acid.

*Zymomonas* was detected in FI 5-day silages, and the inoculation decreased the RA of *Zymononas*, then it disappeared in all 90-day silages. This was attributed to the slower decline of pH in FI silages. It has been reported that ACA is a major inhibitor to *Z. mobilis* because it could destroy membrane integrity and intracellular redox homeostasis [31]. After 90 d, the low pH environment and lack of substrate caused the disappearance of *Zymononas*.

The lower fungal richness and diversity in fresh FI corn than fresh NFI corn were attributed to the infested fungi. However, the trend reversed after ensiling, and NFI silages showed lower fungal richness and diversity than FI silages; this was related to the rapid LA accumulation and pH decline, which inhibited most of the acid-intolerant and aerobic fungi. With the ensiling progress, the pH in all silages decreased to lower levels and further inhibited the activities of more fungi; thus, 90-day silages showed lower fungal community richness and diversity than 5-day silages.

In the PCoA plot, the fresh corn and 5-day silages were clustered together and separated with 90-day silages, and there was a clear separation between FI and NFI of 90-day silages. Similarly, Romero et al. [27] also observed a clear separation and difference in the distribution and structure of the fungal community of oat silage at days 0 and 217, but there was no clear separation among treatments within the same ensiling day.

Little information is available regarding the fungal communities in silage, although the presence and growth of mycotoxigenic fungi have been extensively studied. *Ascomycota*, *Basidiomycota*, and *Chytridiomycota* were three main phyla detected in corn prior to ensiling, and *Ascomycota* was the most predominant phylum. Wang et al. [32] reported that 16 yeast species were isolated from silages, and most of them belonged to the phylum *Ascomycota.*

In the study, *Sarocladium* (17.63%) was the most abundant genus in the FI corn before ensiling, followed by *Candida* (12.83%) and *Penicillium* (9.28%). *Sarocladium* was known as the putative plant-protective endophytes *Sarocladium zeae* (formerly *Acremonium zeae*) from maize, and it may contribute to the inhibition on the growth of infested fungi. *Candida* (34.05%) was the most predominant genus in NFI corn prior to ensiling, followed by *Wickerhamomyces* (19.46%), *Sacocladium* (16.69%), and *Epicoccum* (10.74%). Guan et al. [25] attributed the differences in epiphytic fungal communities to the forage type and climate, because they found that the dominant fungal communities in fresh Napier grass were *Mrakiella*, *Hannaella*, and *Candida*, while Romero et al. [22] reported that the phylum *Ascomycota*, including *Davidiellaceae* (26.9%), *Pleosporaceae* (5.1%), and *Pleosporales* (2.3%), was the dominant fungi phylum in fresh whole-crop oat.

*Issatchenkia* in all silages increased after 90 d of ensiling; it is one of the most frequently described yeasts in different types of silage, and it could assimilate lactic acid and reduce in vitro NDF digestion [33]. Liu et al. [34] also found that *Issatchenkia* was the predominant fungal genus in untreated barley silage at day 60 silages.

In the study, *Candida*, *Wickerhamomyces*, and *Pichia* were detected in all silages. Others have used molecular techniques to confirm that members of *Saccharomycetales,* including *Candida*, *Pichia*, or *Saccharomyces*, are the predominant fungi associated with terminal silage [35]. Most epiphytic fungi rapidly decreased after ensiling because they are obligate aerobes, requiring oxygen to survive; however, *Candida* can thrive under both aerobic and anaerobic conditions. In the study, the higher RA of *Wickerhamomyces* and *Pichia* in FI silages than those of NFI silages was attributed to their antifungal activity. Some strains of *Wickerhamomyces* are known to secrete mycocins with a fungicidal effect; in the study, the fungal infestation in field-grown corn might stimulate the proliferation of *Wickerhamomyces* to antagonize the infested fungi. Some species of *Pichia* have shown a strong biocontrol activity against a variety of molds grown on different cereal grains. Adel Druvefors and Schnurer [36] found that *P. anomala* was unsurpassed among 60 different yeast species in the ability to inhibit the growth of *Penicillium roqueforti* in airtight grain silos.

The RA of *Aspergillus* and *Fusarium* markedly decreased after 90 d of ensiling, and the inoculation expanded this trend irrespective of fungal infestation. The ensiling process inhibited the growth or even eliminated most fungi present at harvest; *Fusarium* spp. are sensitive to low-oxygen and acidic conditions, and their growth is commonly inhibited in silage. Carvalho et al. [37] also reported that mycotoxins are more resistant than mycelia to ensiling conditions, as evidenced by their presence in samples in which the mold can no longer be isolated.

The RA of *Epicoccum* in NFI fresh corn and silages was always lower than that of the corresponding FI fresh corn and silages. *Epicoccum nigrum* is a plant pathogen and endophyte and is a widespread fungus that produces colored pigments against other pathogenic fungi; however, the natural occurrence of mycotoxins produced by this genus in food and feeds has not been studied yet. *Sarocladium* and *Gibberella* were detected in the fresh corn and 5-day silages, and they disappeared after 90 d of ensiling. He et al. [38] reported that the RA of *Sarocladium* was markedly decreased, and propionic acid-treated silages showed the lowest RA of the fungi *Sarocladium* (12.5%) after 140 d of ensiling; it is indicated that *Sarocladium* showed a low tolerance to the acid.

The *Penicillium* in FI silage survived after 90 d of ensiling, while the inoculants decreased the RA of *Penicillium*. *Penicillium verrucosum* and *A. flavus*, belonging to the *Eurotiales,* are known as the main post-harvest mycotoxin producers. *Penicillium expansum*, *P. roqueforti*, and other *Penicillium* spp. are considered to be acidic fungi and tolerant to low oxygen concentrations; thus, they are often observed in terminal silages. Some LAB strains can inhibit fungal growth and the production of mycotoxins, which depend on the production of specific organic acids. Cabo et al. [39] compared the antifungal activity of 56 LAB against four species of *Penicillium*, and found that their antifungal activities were attributed to ACA produced by LAB.

## 4. Conclusions

The fungal infestation was associated with the production of mycotoxins and poor fermentation of corn silages, while inoculants mitigated the adverse effects of fungal infestation on corn silage quality by changing the bacterial and fungal communities. For the farmers, if the corn is infested with rust in the field, the antifungal or detoxified LAB should be applied during the silage making.

## 5. Materials and Methods

### 5.1. Toxigenic Fungi Preparation and Artificial Infestation of Corn in the Field

The toxin-producing fungi *A. flavus* and *F. graminearum*, with the capacity to produce AFB_1_, ZEN, and DON, were selected for artificial infestation on corn during the field growing. *A. flavus* and *F. graminearum* were cultured on potato dextrose agar (PDA) (Bio-way Technology Co., Ltd., Shanghai, China) plates at 30 °C until spores formed. As described by Tefera and Vidal [40], the spores were wet scraped from the culture plate to 50 mL of 0.1% Tween-80 (*v*/*v*) fortified distilled water. To avoid the presence of mycelium, spore suspension was filtered through a sterile cotton filter. We adjusted the prepared spore suspension on a hemocytometer glass slide to a final concentration of 10^6^ cfu/mL.

Corn was grown on the experimental field of Nanjing Agricultural University (32.04° N, 118.88° E, Jiangsu Province, China), which was divided in 6 plots (about 200 m^2^ per plot) and managed in similar conditions. On 27 July 2018, 3 plots of corn at the silking stage were selected randomly for artificial infestation by 5 mL of spore inoculum mixture, which was sprayed on the silks, husks, ears, and leaves. The same plots of corn at the blister stage were artificially re-infested in the same procedure on 10 August 2018.

### 5.2. Silage Preparation and Treatments

Corn plants were harvested on 21 August 2018; 3 plots of corn plants infested with fungal spores (FI) or without fungal spores (NFI) were separately harvested and combined, followed by chopping to a 2–3 cm theoretical length using a forage harvester (93ZT-300; Xingrong Co., Ltd., Guangzhou, China). The FI and NFI forages were separately treated with deionized water (CON, control), or with *L. buchneri* (LB, 1 × 10^6^ cfu/g FW), *L. plantarum* (LT, 1 × 10^6^ cfu/g FW), or *L. buchneri* + *L. plantarum* (BT, both *L. buchneri* and *L. plantarum* applied at 0.5 × 10^6^ cfu/g FW). The treated corn was ensiled in a plastic silo (capacity 5 L, diameter 17.3 cm, height 26.5 cm; Lantian Biological Experimental Instrument Co., Ltd., Jiangsu, China) at a density of 240 kg of DM/m^3^, followed by sealing with two screw tops. The silos were stored at ambient temperature (22 to 28 °C) and five silos per treatment were opened after 5 and 90 d of ensiling.

### 5.3. Sample Preparation and Analysis

Fresh corn was sampled directly after harvesting. Five random silos per treatment were opened after 5 and 90 d of ensiling, and then the ensiled forage was placed into a sterilized plastic container and mixed thoroughly. Representative samples of fresh corn and silage were filled into an envelope and dried at 65 °C for 48 h to a constant weight to measure the DM content. The dried sample was ground with a laboratory pulverizer (FW100, Taisite Instrument Co., Ltd., Tianjin, China), passed through a 1 mm screen, and subjected for determination of CP, WSC, starch, NDF, and ADF concentrations. The concentration of TN was determined using a Kjeldahl nitrogen analyzer (Kjeltec 8400 Analyzer, FOSS Analytical AB, Höganäs, Sweden), and the CP concentration was calculated by multiplying the TN concentration by 6.25. The concentration of WSC was determined by colorimetry after reaction with the anthrone reagent [41]. Concentrations of NDF and ADF were measured sequentially using an ANKOM 200 fiber analyzer (ANKOM Technologies, Macedon, NY, USA) according to Van Soest procedures; heat-stable α-amylase was used in the NDF procedure, and the results were expressed on a DM basis including residual ash.

The concentrations of AFBs, ZEN, DON, and FBs were quantified using a high-performance liquid chromatography-tandem mass spectrometry (UPLC-MS/MS) system with a Sciex QTRAP^®^ 5500 triple quadruple MS/MS (Sciex, Foster City, CA, USA) coupled with an Agilent 1290 binary UHPLC (Agilent Technologies, Waldbronn, Germany). The samples were extracted with 50% acetonitrile aqueous solution, purified by a MycoSpinTM 400 purification column (Romer Labs_ Inc., Union, MO, USA), diluted by the isotope internal standard, and determined by UPLC-MS/MS. Chromatographic separation was performed at 25 °C on a Gemini UPLC C18 column (4.6 mm × 150 mm, 5 μm particle size, Phenomenex, Torrance, CA, USA), and elution was carried out in binary gradient mode with a flow rate of 1000 μL/min; eluent A is 2 mmol/L ammonium acetate added with 0.5% acetic acid in water; eluent B is 2 mmol/L ammonium acetate plus 0.5% ethyl acidic methanol. The following steps were conducted: for the electrospray ionization source, positive ions and negative ions were scanned separately; the ion source temperature in positive ion mode was 650 °C and in negative ion sub-mode was 600 °C; the curtain gas was 35 psi, Gas1 was 60 psi, and Gas2 was 65 psi; the collision gas was medium; the electrospray voltage in positive ion mode was 5000 V, and in negative ion mode was −4500 V.

Sub-samples (20 g) of silage were diluted with 60 mL of distilled water for 24 h at 4 °C; the solution was filtered through 4 layers of gauze and qualitative filter paper (Xinhua Co., Ltd., Hangzhou, China) to obtain the silage extract. Silage pH was immediately measured by a precision pH meter (HANNA pH 211, Hanna Instruments Italia Srl, Padova, Italy). A sub-sample of silage extract was used to measure the ammonia N, which was determined by the phenol-sodium hypochlorite colorimetric method [42]. About 10 mL of the silage extract was centrifuged at 10,000× *g* for 10 min at 4 °C, and the supernatant was reserved for organic acid and ethanol analyses, which were quantified using a high-performance liquid chromatograph system (Agilent HPLC 1260, Agilent Technologies, Inc., Waldbronn, Germany) fitted with a refractive index detector (column: Carbomix^®^H-NP5; Sepax Technologies, Inc., Newark, DE, USA; eluent: 2.5 mmol/L H_2_SO_4_, 0.5 mL/min; temperature: 55 °C).

About 10 g of fresh forage, 5-day, or 90-day silages was mixed with 90 mL of sterile sodium chloride solution (0.85%, 90 mL) at 150 rpm for 2 h. Then, 1 mL of the solution was 10-fold serially diluted for microbial calculations, and the remaining solution was filtered through four layers of medical gauze for DNA extraction. After 48 h of incubation at 37 °C under anaerobic conditions, the number of LAB was counted on deMan, Rogosa, and Sharp agar. The aerobic bacteria (AB) were enumerated on nutrient agar (Bio-way Technology Co., Ltd., Shanghai, China) after 24 h of incubation at 37 °C under aerobic conditions. The total numbers of yeast and molds were counted on potato dextrose agar after 2–3 d of incubation at 28 °C under aerobic conditions. The microbial data were obtained as colony-forming units (cfu) and transformed to a logarithmic scale on a fresh weight (FW) basis.

Three replicates of fresh corn and silages were random chosen and analyzed for bacterial and fungal communities by sequencing the 16S rRNA V3–V4 region and fungal internal transcribed spacer (ITS) region, respectively, using the Illumina MiSeq (Illumina, San Diego, CA, USA) platform. The solution for DNA extraction was centrifuged at 10,000× *g* for 15 min, then the supernatant was discarded, and the pellet was resuspended by the addition of 100 μL of autoclaved Ringers solution. Microbial DNA was extracted using the Fast DNA SPIN kit for soil (MP Biomedicals, Solon, OH, USA) following the protocol provided by the manufacturer. The DNA concentration and purification were evaluated by a NanoDrop 2000 UV-vis spectrophotometer (Thermo Scientific, Wilmington, DE, USA), and DNA quality was checked by 1% agarose gel electrophoresis.

The bacterial 16S rRNA gene V3–V4 region was amplified based on the specific primers 338F (ACTCCTACGGGAGGCAGCAG) and 806R (GGACTACHVGGGTWTCTAAT). The primers ITS1F (5′-CTTGGTCATTTAGAGGAAGTAA-3′) and ITS2aR (5′-GCTGCGTTCTTCATCGATGC-3′) were used to amplify fungal ITS. Then, 10 ng of genomic DNA was amplified using the following PCR conditions: 3 min of denaturation at 95 °C, 27 cycles of 30 s at 95 °C, 30 s for annealing at 55 °C, and 45 s for elongation at 72 °C, and a final extension at 72 °C for 10 min. The resulting PCR products were extracted from a 2% agarose gel, further purified using the AxyPrep DNA Gel Extraction kit (Axygen Biosciences, Union City, CA, USA), and quantified by QuantiFluor™-ST (Promega, Madison, WI, USA) according to the manufacturer’s protocol. The purified PCR amplicons were paired and end sequenced using the Illumina MiSeq PE300 platform (Illumina Inc., San Diego, CA, USA). All raw reads were checked using FLASH (Version 1.2.11), and low-quality sequences (quality scores below 20) were discarded according to the QIIME quality control process (Version 1.7.0). Operational taxonomic units (OTUs) were clustered with a 97% similarity cutoff using UPARSE (Version 7.1, http://drive5.com/uparse/, accessed on 25 June 2021). Then, the chimeric sequences were identified and removed using UCHIME. The community structure was analyzed at the phylum and genus levels using the Silva database (Release128, http://www.arb-silva.de, accessed on 25 June 2021) with a confidence threshold of 70%. To visualize the degree of bacterial and fungi composition similarity between samples, principal coordinate analysis (PCoA) was conducted on the OTU-based unweighted principal coordinate analysis. The sequencing data were analyzed on the free online platform of Majorbio I-Sanger Cloud Platform (www.i-sanger.com, accessed on 25 June 2021).

### 5.4. Statistical Analysis

The data of fermentation quality and mycotoxins were analyzed as a completely randomized factorial design in a 2 (with or without infestation) × 4 (inoculants: deionized water; *L. buchneri*; *L. plantarum*; *L. buchneri* + *L. plantarum*) factorial arrangement of treatments. A model containing fungal infestation, inoculants, and their interactions was used to analyze the data using the GLM procedure of SAS (version 9.3; SAS Institute Inc., Cary, NC, USA).

The data of bacterial and fungal diversity were analyzed as a completely randomized factorial design in a 2 (with or without infestation) × 2 (5 and 90 d of ensiling) × 4 (inoculants: deionized water; *L. buchneri*; *L. plantarum*; *L. buchneri* + *L. plantarum*) factorial arrangement of treatments. A model containing fungal infestation, ensiling days, inoculants, and their interactions was used to analyze the data using the GLM procedure of SAS (version 9.3; SAS Institute Inc., Cary, NC, USA).

Data for the effect of fungal infestation or additives were determined by Tukey’s multiple comparison only if there were no significant interactions (*p* > 0.05) and if significance was detected for the specific effect (*p* ≤ 0.05). When a significant interaction was detected (*p* ≤ 0.05), the means were tested using Tukey’s test.

## Figures and Tables

**Table 1:** Microbial populations (log_10_ cfu/g FW basis) and chemical composition (g/kg DM basis unless stated otherwise) of fresh corn.

Items	DM g/kg FW	WSC	CP	NDF	ADF	Starch	LAB	Yeasts and Molds	AB	Enterobacteria
FI corn	339	171	45.3	486	243	105	7.50	6.80 ^a^	8.37	6.17
NFI corn	333	177	50.7	567	203	112	7.20	4.37 ^b^	7.93	6.57

^a,b^ Means within a row with different superscripts letters are significantly different (*p* < 0.05). FI, fungal infestation; NFI, non-fungal infestation. FW, fresh weight; DM, dry matter; WSC, water-soluble carbohydrates; CP, crude protein; CFU, colony-forming units; LAB, lactic acid bacteria; AB, aerobic bacteria; NDF, neutral detergent fiber; ADF, acid detergent fiber.

**Table 2:** Effect of fungal infestation and inoculants on the pH, WSC, fermentation products (g/kg DM basis), and microbial populations (log_10_ cfu/g FW basis) of corn silage on day 90.

Item		pH	Lactic Acid	Acetic Acid	Latic/ Acetic Acid	Ethanol	WSC	Ammonia N g/kg TN	LAB	AB	Yeasts
FI	CON	3.66 ^a^	88.2 ^bc^	29.0 ^ab^	3.04 ^c^	23.3	38.1	115 ^a^	3.87	0.00 ^a^	1.30
	LB	3.64 ^ab^	95.1 ^abc^	30.8 ^a^	3.11 ^c^	23.5	33.0	110 ^ab^	3.25	2.43 ^a^	0.00
	LT	3.62 ^b^	88.6 ^bc^	26.7 ^b^	3.32 ^c^	22.5	39.1	104 ^ab^	2.87	1.67 ^a^	0.77
	BT	3.61 ^b^	98.3 ^abc^	27.1 ^b^	3.62 ^c^	23.4	36.4	100 ^b^	3.20	0.00 ^a^	0.33
NFI	CON	3.57 ^c^	87.3 ^c^	16.0 ^c^	5.45 ^b^	24.4	41.8	71.1 ^c^	2.98	1.60 ^a^	0.33
	LB	3.53 ^d^	106 ^a^	16.1 ^c^	6.58 ^a^	20.5	32.2	71.9 ^c^	2.93	1.10 ^a^	0.78
	LT	3.50 ^e^	105 ^a^	15.5 ^c^	6.82 ^a^	18.2	31.2	74.3 ^c^	2.97	0.00 ^a^	0.77
	BT	3.44 ^f^	104 ^ab^	16.0 ^c^	6.52 ^a^	18.1	30.4	66.9 ^c^	2.53	1.67 ^a^	0.00
Fungal infestation means											
FI		3.63 ^a^	92.5 ^b^	28.4 ^a^	3.27 ^b^	23.2 ^a^	36.6	107 ^a^	3.30	1.03	0.60
NFI		3.51 ^b^	101 ^a^	15.9 ^b^	6.34 ^a^	20.3 ^b^	33.9	71.0 ^b^	2.85	1.09	0.47
Inoculant means											
CON		3.61 ^a^	87.8 ^b^	22.5 ^ab^	4.25 ^b^	23.8	40.0 ^a^	93.3 ^a^	3.43	0.80	0.82
LB		3.54 ^c^	100 ^a^	23.5 ^a^	4.84 ^a^	22.0	32.6 ^b^	90.9 ^ab^	3.09	1.77	0.39
LT		3.58 ^b^	96.9 ^ab^	21.1 ^b^	5.07 ^a^	20.4	35.1 ^ab^	89.1 ^ab^	2.92	0.83	0.77
BT		3.56 ^c^	101 ^a^	21.5 ^ab^	5.07 ^a^	20.8	33.4 ^ab^	83.4 ^b^	2.87	0.83	0.17
SEM		0.015	1.83	1.35	0.334	0.66	1.08	3.98	0.400	0.274	0.145
Effects and interactions											
Fungal infestation		<0.01	<0.01	<0.01	<0.01	0.02	0.14	<0.01	0.64	0.89	0.65
Inoculants		<0.01	<0.01	0.03	<0.01	0.12	0.04	0.01	0.97	0.44	0.32
Fungal infestation × inoculants		<0.01	0.09	0.09	<0.01	0.20	0.12	0.07	0.98	0.04	0.20

^a–f^ Means in columns within a category with unlike superscripts differ (*p* < 0.05).FI, fungal infestation; NFI, non-fungal infestation; CON, control; LB, *L. buchneri* applied at 1 × 10^6^ cfu/g FW; LT, *L. plantarum* applied at 1 × 10^6^ cfu/g FW; BT, *L. buchneri* and *L. plantarum* applied at 0.5 × 10^6^ cfu/g FW. SEM, standard error of means. FW, fresh weight; DM, dry matter; WSC, water-soluble carbohydrates; TN, total nitrogen; LAB, lactic acid bacteria; AB, aerobic bacteria.

**Table 3:** Effect of fungal infestation and inoculants on mycotoxin concentrations (μg/kg DM basis) of corn silage after 90 d of ensiling.

Item		AFB_1_	AFB_2_	ZEN	DON	FB_1_	FB_2_	FB_3_
FI	Fresh	35.1 ^a^	2.77	2015 ^a^	34.8 ^a^	999 ^a^	265	20.3
	CON	21.3 ^b^	0.00	1598 ^b^	17.4 ^b^	376 ^bc^	259	60.0
	LB	11.2 ^c^	4.33	1518 ^b^	6.12 ^c^	173 ^bc^	91.7	47.7
	LT	13.4 ^bc^	2.90	1563 ^b^	6.70 ^c^	126 ^c^	152	68.3
	BT	7.13 ^cd^	<0.50	1094 ^c^	4.51 ^c^	207 ^bc^	148	79.3
NFI	Fresh	<0.50	<0.50	406 ^d^	21.5 ^b^	467 ^b^	129	48.7
	CON	<0.50	<0.50	449 ^d^	3.05 ^c^	266 ^bc^	60.3	18.0
	LB	<0.50	<0.50	405 ^d^	5.48 ^c^	366 ^bc^	67.3	20.7
	LT	<0.50	<0.50	488 ^d^	3.17 ^c^	246 ^bc^	79.3	27.0
	BT	<0.50	<0.50	335 ^d^	4.73 ^c^	210 ^bc^	72.0	17.7
Infestation means (90 days)								
FI		13.3	1.81	1443 ^a^	8.68 ^a^	221 ^b^	163 ^a^	63.8 ^a^
NFI		<0.50	<0.50	419 ^b^	4.11 ^b^	272 ^a^	69.8 ^b^	20.8 ^b^
Inoculants means (90 days)								
CON		10.7 ^a^	0.00	1023 ^a^	10.2 ^a^	321 ^a^	160	39.0
LB		5.62 ^b^	2.17	962 ^a^	5.80 ^b^	270 ^ab^	79.5	34.2
LT		6.72 ^ab^	1.45	1026 ^a^	4.93 ^b^	186 ^b^	116	47.7
BT		3.57 ^b^	0.00	714 ^b^	4.62 ^b^	208 ^b^	110	48.5
SEM		2.130	0.421	113.9	1.871	47.9	17.27	5.33
Effects and interactions								
Fungal infestation		<0.01	0.06	<0.01	<0.01	0.03	<0.01	<0.01
Inoculants		<0.01	0.27	<0.01	<0.01	<0.01	0.27	0.71
Fungal infestation × inoculants		<0.01	0.27	0.03	<0.01	<0.01	0.18	0.70

^a–d^ Means in columns within a category with unlike superscripts differ (*p* < 0.05). FI, fungal infestation; NFI, non-fungal infestation; CON, control; LB, *L. buchneri* applied at 1 × 10^6^ cfu/g FW; LT, *L. plantarum* applied at 1 × 10^6^ cfu/g FW; BT, *L. buchneri* and *L. plantarum* applied at 0.5 × 10^6^ cfu/g FW. SEM, standard error of means; FW, fresh weight; DM, dry matter.

**Table 4:** The α-diversity indices of bacterial and fungal communities of corn silage.

Treatment	Bacterial Community		Fungi Community	
	Shannon	Chao 1	Shannon	Chao 1
FI Fresh	3.80	427	2.33 ^b^	148 ^b^
NFI Fresh	3.22	490	3.00 ^a^	272 ^a^
SEM	0.165	19.2	0.157	29.0
*p*-value	0.07	0.10	<0.01	<0.01
Fungal infestation				
FI	2.29 ^a^	258	2.55 ^a^	168
NFI	1.58 ^b^	280	1.50 ^b^	166
Inoculants				
CON	1.98	307 ^a^	2.08	177
LB	2.03	270 ^ab^	2.02	160
LT	1.90	229 ^b^	2.1	160
BT	1.82	271 ^ab^	1.89	172
Days				
5	1.52 ^b^	194 ^a^	2.68 ^a^	228 ^a^
90	2.35 ^a^	345 ^b^	1.36 ^b^	107 ^b^
SEM	0.095	14.7	0.154	10.5
*p*-value				
Fungal infestation	<0.01	0.15	<0.01	0.83
Inoculants	0.35	<0.01	0.32	0.50
Days	<0.01	<0.01	<0.01	<0.01
Fungal infestation × inoculants	0.07	0.05	0.09	0.57
Fungal infestation × days	<0.01	<0.01	<0.01	<0.01
Inoculants × days	0.2063	0.30	0.18	0.29
Fungal infestation × inoculants × days	0.6783	0.11	0.12	0.24

^a,b^ Means in columns within a category with unlike superscripts differ (*p* < 0.05). FI, fungal infestation; NFI, non-fungal infestation; CON, control; LB, *L. buchneri* applied at 1 × 10^6^ cfu/g FW; LT, *L. plantarum* applied at 1 × 10^6^ cfu/g FW; BT, *L. buchneri* and *L. plantarum* applied at 0.5 × 10^6^ cfu/g FW. SEM, standard error of means.

## Data Availability

The datasets generated for this study can be found in Sequence Read Archive under BioProject, PRJNA 741827 (https://www.ncbi.nlm.nih.gov/sra, accessed on 25 June 2021).
